# High-flow nasal cannula oxygen therapy for admitted COPD-patients. A retrospective cohort study

**DOI:** 10.1371/journal.pone.0272372

**Published:** 2022-10-05

**Authors:** Pieter Veenstra, Nic J. G. M. Veeger, Ralph J. H. Koppers, Marieke L. Duiverman, Wouter H. van Geffen

**Affiliations:** 1 Department of Respiratory Medicine, Medical Center Leeuwarden, Leeuwarden, Netherlands; 2 Department of Epidemiology, University of Groningen, University Medical Center Groningen, Groningen, Netherlands; 3 Department of Pulmonary Diseases/Home Mechanical Ventilation, University of Groningen, University Medical Center Groningen, Groningen, Netherlands; Stanford University School of Medicine, UNITED STATES

## Abstract

**Background:**

The use of High-flow nasal cannula (HFNC) is increasing in admitted COPD-patients and could provide a step in between non-invasive ventilation (NIV) and standard oxygen supply. Recent studies demonstrated that HFNC is capable of facilitating secretion removal and reduce the work of breathing. Therefore, it might be of advantage in the treatment of acute exacerbations of COPD (AECOPD). No randomized trials have assessed this for admitted COPD-patients on a regular ward and only limited data from non-randomized studies is available.

**Objectives:**

The aim of our study was to identify the reasons to initiate treatment with HFNC in a group of COPD-patients during an exacerbation, further identify those most likely to benefit from HFNC treatment and to find factors associated with treatment success on the pulmonary ward.

**Material and methods:**

This retrospective study included COPD-patients admitted to the pulmonary ward and treated with HFNC from April 2016 until April 2019. Only patients admitted with severe acute exacerbations were included. Patients who had an indication for NIV-treatment where treated with NIV and were included only if they subsequently needed HFNC, e.g. when they did not tolerate NIV. Known asthma patients were excluded.

**Results:**

A total of 173 patients were included. Stasis of sputum was the indication most reported to initiate HFNC-treatment. Treatment was well tolerated in 83% of the patients. Cardiac and vascular co-morbidities were significantly associated with a smaller chance of successful treatment (Respectively OR = 0.435; p = 0.013 and OR = 0.493;p = 0.035). Clinical assessment judged HFNC-treatment to be successful in 61% of the patients. Furthermore, in-hospital treatment with NIV was associated with a higher chance of HFNC failure afterwards (OR = 0.439; p = 0.045).

**Conclusion:**

This large retrospective study showed that HFNC-treatment in patients with an AECOPD was initiated most often for sputum stasis as primary reason. Factors associated with improved outcomes of HFNC-treatment was the absence of vascular and/or cardiac co-morbidities and no need for in-hospital NIV-treatment.

## Introduction

Chronic Obstructive Pulmonary Disease (COPD) patients often suffer from periods of acute worsening of shortness of breath. During severe exacerbations, patients need admission to a hospital [1]. Additional oxygen supply is a cornerstone of the in-hospital treatment of these patients. However, standard oxygen therapy via nasal prongs is limited by a low fraction of inspired oxygen (FiO_2_) due to a limited flow rate of usually cold and dry air [2–4]. In addition standard oxygen therapy does not influence mucus extraction.

Moreover, in patients with acute acidotic hypercapnic respiratory failure, non-invasive ventilation (NIV) is a proven effective therapy. It reduces the work of breathing, improves gas exchange, reduces the length of hospital stay, decreases the need for endotracheal intubation and improves mortality [5–8]. However, patients may not always tolerate NIV-treatment due to discomfort [8, 9]. Also talking, eating, and especially mucus expectoration, a common problem during COPD-exacerbations, are difficult during NIV-treatment.

If NIV fails, in clinical practice usually two options remain, step-up to invasive ventilation or accept to step-down to standard oxygen therapy. If hypercapnic respiratory failure persists, invasive ventilation is recommended. However, invasive ventilation in COPD-patients is associated with worse outcomes, prolonged hospitalization and mortality as compared to NIV [10]. Next to its limited effect on mucus, standard oxygen therapy will not treat hypercapnic respiratory failure, and may even worsen hypercapnia in some cases.

To address these problems, an alternative treatment option positioned in between NIV and standard oxygen could be of additional value. Potentially, such a treatment could be used to treat COPD-patients with more severe hypoxemic respiratory failure needing more than standard oxygen therapy and patients with a mild to moderate combined hypoxemic/hypercapnic respiratory failure who do not tolerate NIV, especially those with mucus-related problems. Treatment with high-flow nasal cannula (HFNC) could be considered as this step in between standard oxygen and NIV. With HFNC heated and humidified air optionally supplemented with additional oxygen can be delivered at high flow rates up to 60L/min and this may have multiple advantages for the treatment of COPD-exacerbations.

First, the humidification and heating of the delivered air may facilitate secretion removal, reduce airway inflammation and avoid epithelial injury [11], and potentially preventing bronchospasm [12]. Secondly, the high flow provides some positive airway pressure; it increases nasopharyngeal airway pressure that peaks at the end of expiration, which may counteract on the intrinsic positive end-expiratory pressure (PEEPi) and thus may decrease the work of breathing [13–16]. Although the effect of PEEP depends on mouth close of the patient. Furthermore with the high flow of HFNC the generally present higher inspiratory (flow) demand in COPD-patients during exacerbations can be met. Finally, The high flow of oxygen of HFNC reduces dead space. The combination of these mechanisms of HFNC has been shown to reduce P_a_CO2 [17–19].

While awaiting large randomized controlled trials, HFNC is already being used in clinical practice for COPD-patients. From these clinical practices, valuable information can be obtained with regard to reasons for initiating treatment with HFNC in COPD-patients with an exacerbation and selection of patients that benefit most from treatment. We retrospectively analyzed data of admitted COPD-patients treated with HFNC at a pulmonary ward in order to address these questions. Furthermore, we aimed to describe clinical outcomes with regard to used settings of the HFNC, treatment tolerance and factors associated with successful treatment.

## Methods

### Study population

We performed a retrospective study including COPD patients (>18 years) admitted with a COPD exacerbation to the pulmonary ward of a Dutch teaching hospital and treated with HFNC in the period of April 2016 until April 2019. The definition of a COPD-exacerbation by the Global initiative for chronic obstructive lung diseases was used [1, 20]. Only patients with exacerbation of COPD as primary reason for admission and treatment were included. Patients who had an indication for NIV-treatment where treated with NIV first and were included only if they subsequently received HFNC. Only ward-based HFNC was analyzed; treatment periods at an Intensive care unit (ICU) were not included, but step down treatments at the pulmonary ward after ICU-treatment however were included. Patients who were admitted and treated with HFNC multiple times were included only once. Patients with asthma, known active lung malignancy, cerebrovascular accident and/or myocardial infarction less than three months ago, neuromuscular disease and hemodialysis were excluded from the analysis.

### Data recorded

Data were retrieved from the hospital-electronic patient systems. Demographic data such as age, gender, COPD GOLD-stage, presence of co-morbidities (as recorded in medical history), stable state lung function (forced expiratory volume in 1 second (FEV1)) and concomitant treatments were recorded. Recording of co-morbidities was limited to other lung diseases (such as bronchiectasis, lung embolus, interstitial lung disease a.o.) cardiac- (myocardial infarction, heart failure or arrhythmias), vascular- (hypertension, peripheral arterial disease), neurologic co-morbidities and/or diabetes. Also, the number of COPD exacerbations for which treatment with antibiotics and/or corticosteroids was required in the year before admission was recorded, as well as the number of admissions in the previous year.

Vital signs, such as respiratory rate (RR), heart rate (HR) and oxygen saturation (SO_2_) were retrieved from 3 different time points: before the start of HFNC-treatment (obtained within 24 hours before the start), during HFNC-treatment, and within 24 hours after ending HFNC-treatment. Arterial blood gasses (ABG) values were used for analysis if obtained within 48 hours before and 48 hours after HFNC-treatment. The settings of the HFNC-treatment; (inspired oxygen fraction (FiO_2_), flow rate and temperature of the humidified air), were recorded at start and during treatment. We assessed the need for NIV or invasive ventilation, length of hospital stay and mortality.

### Treatment

The decision to initiate HFNC was made by the treating pulmonologist based on clinical arguments. Every included patient was treated with the same device; (AIRVO® Humidification system; Fisher & Paykel healthcare, Auckland, New Zealand). The HFNC was set according to decision of the treating physician.

### Outcome assessment

The judgement whether HFNC was successful or not was made by the attending consultant respiratory medicine based on the clinical examination of the patient, independent of the study team. Treatment was deemed non-successful when patients showed no clinical improvement, or did not tolerate the treatment. Successful treatment was defined as the absence of treatment failure.

### Statistical analysis

Descriptive data were expressed as mean ± standard deviation or as percentage (%). The distribution of data was assessed by performing a Shapiro-Wilk test and examining a histogram and Q-Q plot for every independent value. Median and interquartile ranges (Q1 and Q3) were used for non-normal distributed data and percentage (%) was used for categorical data.

A Chi-square or Fisher’s-exact test was used to compare nominal/categorical variables. When comparing values from the same patient a paired T-test was used when the data was normally distributed and a sign-test when the data was non-normally distributed.

For comparison between groups of continuous variables that were normally distributed an unpaired T-test was used. When these values were non-normally distributed a Mann Whitney U-test was used to calculate significance. All tests were two sided and considered significant if the P-value was below 0.05. Analysis was performed using SPSS statistics version 24.

For comparison of factors associated with treatment success a multiple logistic regression was performed. First of a univariate analysis was done for all the variables, next the variables which were shown to have a slight association (p<0.15) were taken into account in the multiple regression analysis. Backward selection was used to further narrow down the variables. The variables were considered statistically significant if the P-value was below 0.05.

### Ethics

The Medical Ethics Committee of our institution (RTPO Leeuwarden) confirmed the conduct of this retrospective study and the institutional board approved the execution of the study without the need for consent in accordance with Dutch regulations (ID NMWMO356). All data was handled confidentially and anonymously.

## Results

### Baseline characteristics

**Fig 1:**
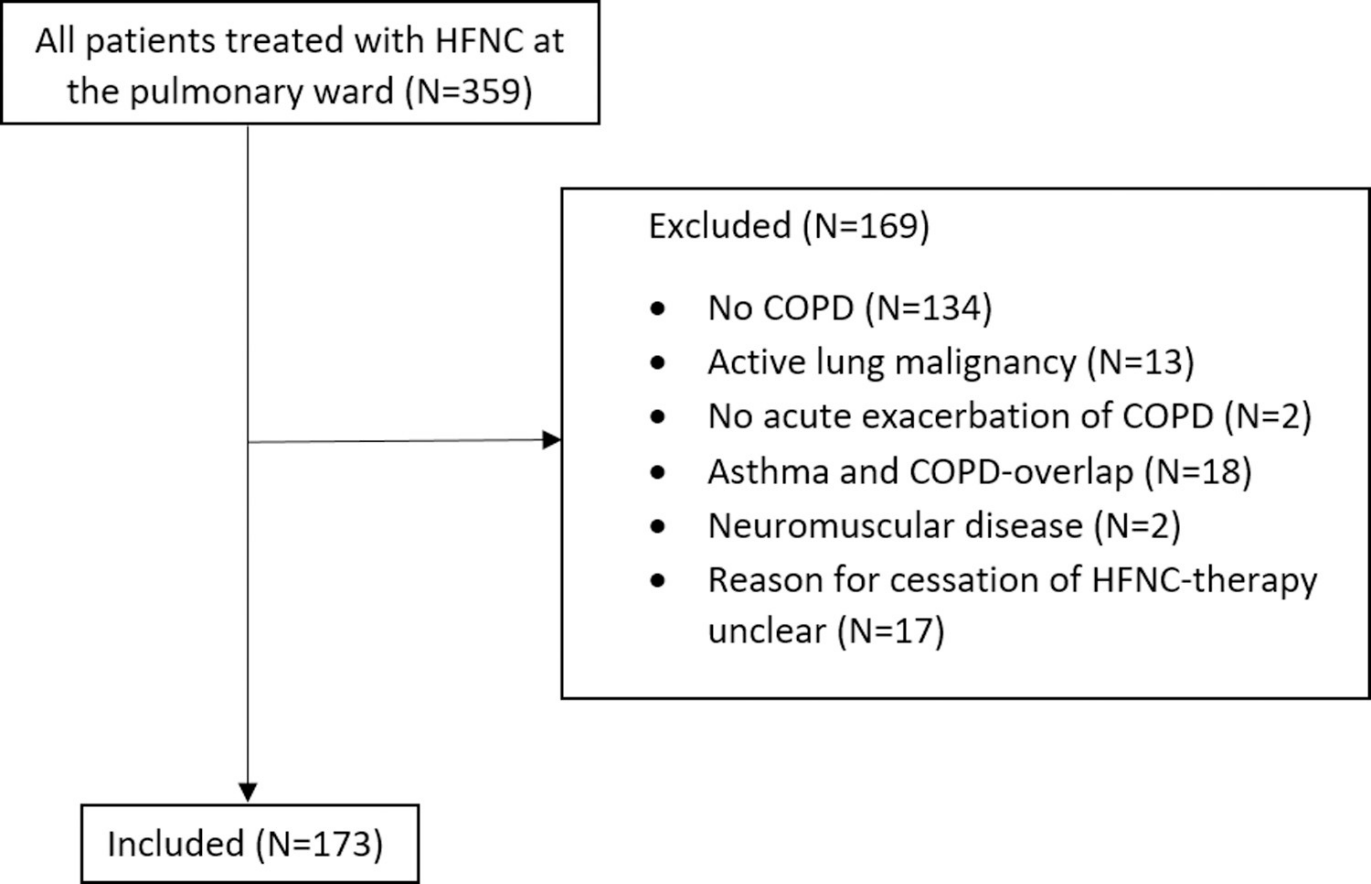
Flowchart of all included patients.

Among all the patients treated with HFNC at the pulmonary ward, 173 patients met the inclusion criteria (Fig 1). The characteristics of the included patients are summarized in Table 1. The mean age of the patients was 71 (± 10) years and 87 of the patients (51%) were male. Most of the patients were former smokers (65%) and had a COPD GOLD classification II (37%) or III (35.8%) with a mean FEV_1_ of 1.19L (46% of predicted). Almost half of the patients were known with cardiac (48.6%) and/or vascular (54.9%) co-morbidities. The patients had a median exacerbation rate of 1.00 (0–12) exacerbations in the previous year with 0 (0–6) admissions in the previous year.

**Table 1:** Baseline characteristics of the included patients.

Characteristic	N	Mean ± St. dev./Percentage
Age (years)	173	71 ± 10
Man	87	51%
Weight (kg)	172	75.3 ± 20.5
Length (cm)	172	171 ± 9
BMI (kg/m^2^)	172	25.8 ± 6.4
**Smoking**	**173**	
Current	51	29.5%
Former	113	65.3%
Never	9	5.2%
**COPD Gold stage for Airway obstruction**	**166**	
I	4	2.3%
II	64	37.0%
III	62	35.8%
IV	36	20.8%
**Comorbidities:**		
Other Lung diseases	40	23.3%
Cardiac	84	48.6%
Vascular	95	54.9%
Neurologic	55	31.8%
Diabetes	35	20.2%
**Stable state Lung function:**	**162**	
FEV1 (L)	162	1.19 ± 0.49
VCmax (L)	162	2.77 ± 0.95
FEV1 (% predicted)	162	46 ± 17
FEV1/VCmax (%)	162	44 ± 13
TLco (% predicted)	88	47 ± 16
**Current maintenance treatment at home**		
Long-acting β_2_-agonists	140	80.9%
Long-acting muscarinic antagonists	121	69.9%
Inhalated corticosteroids	132	76.3%
Oral Corticosteroids	41	23.7%
Antibiotics	36	20.8%
Oxygen treatment at home	33	19.1%
**Year before treatment:**		
Number of exacerbations in which treatment was needed	173	1.00 (0–3)
Number of admissions	173	0.00 (0–1)
**First arterial blood gas values:**		
pH	101	7,39 (± 0,07)
pCO_2_ (kPa)	101	6,82 (± 2,01)
pO_2_ (kPa)	101	8,15 (± 2,01)

Values shown are mean ± standard deviation or percentage (%) for categorical data.

N: number of patients; BMI: Body mass index; COPD: Chronic obstructive pulmonary disease; FEV1 forced expiratory volume in 1 second; L: litre; VCmax: maximal vital capacity; FEV1/VCmax: Tiffeneau index; TLco: diffusing capacity for carbon monoxide.

In addition to the HFNC-treatment, 20.2% of the patients needed treatment with NIV during the hospital admission and 80.3% received treatment with different means of O_2_-suppletion (Table 2).

**Table 2:** Concomitant in-hospital treatment.

Concomitant in-hospital treatment:	N	Percentage
SABA/SAMA aerosol via nebulizer	165	95.4%
Oral corticosteroids	163	94.2%
Antibiotics	133	76.9%
O_2_-suppletion	139	80.3%
NIV	35	20.2%

In-hospital treatment in addition to treatment with HFNC for all included patients. Values shown are the number of patients (N) and percentage (%). SABA: short-acting β-agonist; SAMA: short-acting muscarinic antagonist; NIV: Non-invasive ventilation.

### HFNC-settings

HFNC-settings were recorded properly in the majority of the treated patients at start of treatment. The median flow rate used was 30 L/min (N = 156, IQR: Q1 30L/min, Q3 40L/min), with a median FiO_2_ of 25% (N = 164, IQR: Q1 21%, Q3 32%) and a median temperature of 31°C (N = 147, IQR: Q1 31°C, Q3 31°C).

### Reasons to start HFNC-treatment

**Fig 2:**
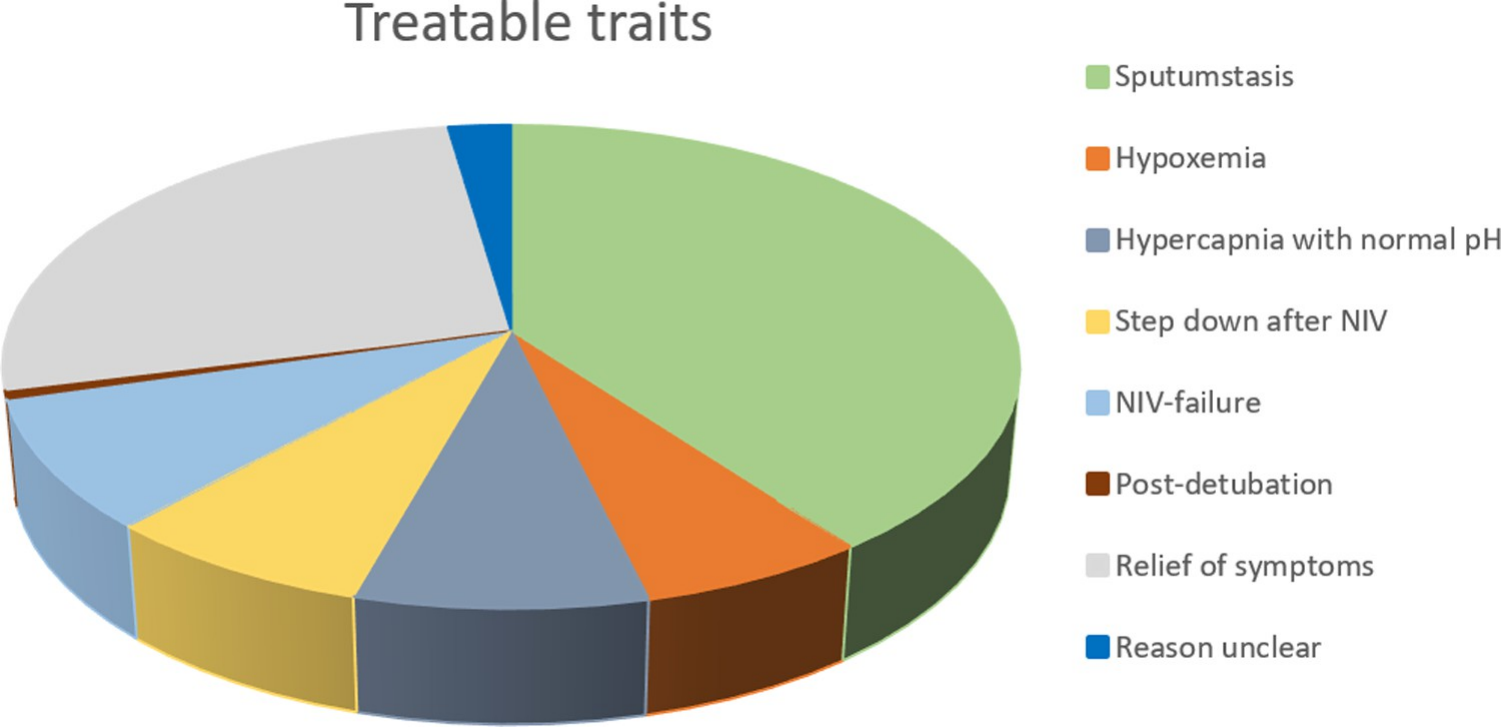
Treatable traits for HFNC in included COPD-patients.

In 69 patients (39.9%), the primary reason for treatment was stasis of sputum. Eleven patients (6.4%) were treated because of hypoxemia despite regular oxygen suppletion and 14 patients (8.1%) because of hypercapnia with normal pH. In 13 patients (7.5%) HFNC-treatment was a step-down after NIV-treatment, in 15 patients (8.7%) HFNC-treatment was initiated after NIV-treatment failed, mostly because NIV-treatment was not tolerated and in only 1 patient (0.6%) HFNC-treatment was initiated after detubation. In 46 patients (26.6%) HFNC was started for relief of symptoms related to dyspnea and in 4 patients (2.3%) the primary reason for starting treatment was not retrievable (Fig 2).

### Outcomes

#### Vital signs

The heart rate decreased after the start of treatment, (Improvement before-during: 4.0/min, p = 0.005) and remained lower after treatment (Improvement before-after: 5.3/min, p = <0.001). SO_2_ was increased after treatment with HFNC (Improvement before-after: -0.8%, p = 0.033) as well as the respiratory rate (Improvement before-after: 1.3, p = 0.001) (Table 3).

**Table 3:** Mean values before, during and after treatment with the HFNC.

	Before HFNC-treatment		During HFNC-treatment					After HFNC-treatment				
	N		N		NΔ	Improvement Before-During	P-value	N		NΔ	Improvement Before-After	P-value
**Vital signs**												
Respiratory rate (/min)	172	21 ± 5	142	20 ± 4	141	0.5 (-0.11 to 1.16)	0.10	173	19 ± 4	172	1.3 (0.5 to -0.2)	0.001
Heart rate (/min)	172	90 ± 19	142	87 ± 15	141	4.0 (1.2 to 6.8)	0.005	170	85 ± 15	169	5.3 (2.7–7.8)	<0.001
Saturation (%)	172	92 ± 5	145	92 ± 4	144	- 0.3 (-1.0 to 0.4)	0.42	173	92 ± 4	172	- 0.8 (-1.6 to -0.1)	0.033

Values shown are mean and standard deviation. For Δ-values (Δ Before-During and Δ Before-After) the mean change and 95% confidence interval for the means are shown. A paired T-test was used for comparison between values before and during and before and after.

N: number of patients; NΔ: the number of patients with known values before and during or before and after.

#### Outcome of HFNC-treatment

**Fig 3:**
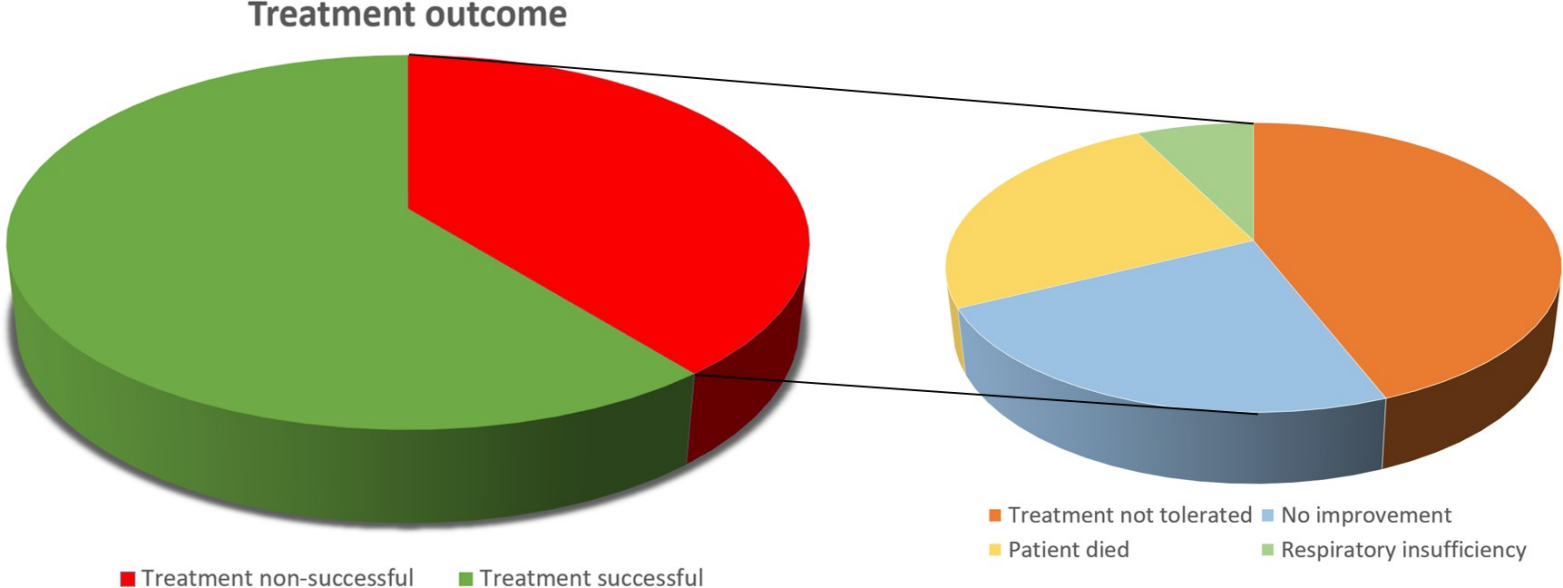
Treatment outcome of HFNC-treatment.

The mean length of hospital stay for all patients was 9.5 ± 6.2 days. Treatment outcome is shown in Fig 3. In 105 patients (61%), treatment was successful and in 68 patients (39%) treatment was non-successful. In 5 patients (3%) treatment was non-successful due to respiratory insufficiency, these patients were initiated on NIV or were intubated. Sixteen patients (9%) showed no improvement, 30 patients (17%) did not tolerate HFNC-treatment and 17 patients (10%) died.

#### Factors associated with success and failure

Comparing the patients that were treated successfully to those who were not (Table 4), there were no differences in stable state lung function, number of exacerbations and admissions due to exacerbations in the previous year, maintenance treatment at home and in-hospital treatment. Patients with treatment success had significant fewer cardiac and vascular co-morbidities than the patients with treatment failure (cardiac co-morbidities 41% vs. 60%, p = 0.02 and vascular co-morbidities 48% vs. 66%, p = 0.02.

**Table 4:** Comparison between patients whom had successful treatment or wit treatment failure.

Patient characteristics	N	Treatment Successful (N = 105)	N	Treatment failure (N = 68)	P-value
Age, mean (years)	105	70 ± 10	68	72 ± 10	0.45
Gender, man	105	45%	68	59%	0.072
Weight, mean (kg)	105	74 ± 18	67	79 ± 23	0.13
Length, mean (cm)	105	170 ± 8	67	172 ± 10	0.12
BMI, mean (kg/m^2^)	105	25.5 ± 6.0	67	26.4 ± 7.1	0.37
**Smoking**					0.17
Current	26	24.8%	25	36.8%	
Former	72	68.6%	41	60.3%	
Never	7	6.7%	2	2.9%	
**COPD Gold stage for Airway obstruction**					0.81
I	3	2.9%	1	1.5%	
II	36	34.3%	28	41.2%	
III	39	37.1%	23	33.8%	
IV	22	21.0%	14	20.6%	
**Co-morbidities:**					
Other lung disease	25	23.8%	21	30.9%	0.38
Cardiac	43	41.0%	41	60.3%	0.02*
Vascular	50	47.6%	45	66.2%	0.02*
Neurologic	29	27.6%	26	38.2%	0.18
Diabetes	19	18.1%	16	23.5%	0.44
**Year before treatment**					
Number of exacerbations in which treatment was needed	105	1.00 (0–2)	68	1.00 (0–3)	0.08
Number of admissions	105	0.00 (0–1)	68	0.00 (0–1)	0.12
**Reason for HFNC-treatment**					0.71
Hypoxemia	9	8.6%	2	2.9%	
Sputum stasis	42	40.0%	27	39.7%	
Hypercapnia with normal pH	8	7.6%	6	8.8%	
Step-down after NIV	7	6.7%	6	8.8%	
NIV Failure	7	6.7%	8	11.8%	
Post-detubation	1	1.0%	0	0%	
Relief of Dyspnea	29	27.6%	17	25.0%	
Reason unclear	2	1.9%	2	2.9%	
**Values before HFNC-treatment**					
Respiratory rate, mean (/min)	105	20.4 ± 4.2	67	20.9 ± 5.0	0.48
Heart rate, mean (/min)	104	88.7 ± 18.3	68	93.2 ± 19.3	0.13
Saturation, mean (%)	104	91.6 ± 4.2	68	91.3 ± 5.2	0.70
**Values during HFNC-treatment**					
Respiratory rate, mean (/min)	97	19.9 ± 3.5	45	21.2 ± 3.6	0.038*
Heart rate, mean (/min)	97	84.9 ± 13.3	45	90.5 ± 18.0	0.038*
Saturation, mean (%)	98	92.0 ± 3.2	47	90.5 ± 4.6	0.044*
**Values after HFNC-treatment**					
Respiratory rate, mean (/min)	105	18.6 ± 2.9	68	20.3 ± 4.4	0.006*
Heart rate, mean (/min)	105	81.4 ± 13.1	65	91.1 ± 16.3	0.000*
Saturation, mean (%)	105	93.2 ± 3.2	68	91.1 ± 5.6	0.007*
Time on the HFNC, median (hours)	101	71 (45–122)	49	26 (13–75)	0.000*
**Secondary outcomes**					
Time of Hospital stay, median (days)	105	8 (6–12)	68	7 (5–11)	0.059
Progression to NIV	1	1.0%	4	5.9%	0.079
Progression to intubation	0	0%	2	2.9%	0.15
Progression to O_2_ at home	9	8.6%	4	5.9%	0.25
Mortality	17	16.2%	31	45.6%	<0.000*
During hos- pital stay	1	1.0%	18	26.5%	<0.000*
90 days	6	5.7%	20	29.4%	0.066
After 90 days	11	10.5%	11	16.2%	0.35

Mean ± standard deviation are shown for normally distributed data. Median (minimum and maximum) is used for non-normal distributed data and percentage (%) was used for categorical data. For every independent variable the number of patients are shown (N).

N: number of patients; BMI: Body mass index; COPD: Chronic obstructive pulmonary disease; AECOPD: acute exacerbation of COPD; NIV: non-invasive ventilation.

Vital signs at the start of HFNC-treatment did not differ significantly. During and after HFNC-treatment, patients treated successfully showed significant lower respiratory rates (During; 20/min vs. 21/min, p = 0.038, After; 19/min vs. 20/min, p = 0.006) and a significant higher SO_2_ (During; 92% vs. 91%, p = 0.044, After; 93 vs. 91, p = 0.007).

No significant difference between the two groups was observed in PaCO_2_, PaO_2_ and pH before, during or after HFNC-treatment. Patients treated successfully were treated longer on average than the non-successful treatment group (median 71 hours vs. 26 hours, p<0.001). There was no difference observed between patients treated non-successfully and patients treated successfully in intubation rate or progression to O_2_-treatment at home, although numbers for analysis were small (Table 4).

In the multivariate logistic regression analysis, the presence of cardiac and vascular co-morbidities and in-hospital treatment with NIV were independently associated with a lower HFNC success rate (Respectively OR = 0,435, OR = 0,493 and OR = 0,439, see Table 5).

**Table 5:** Multivariate logistic regression analysis.

Patient characteristics	N	Odds Ratio	P-value	Lower bound (95%)	Upper bound (95%)
**Co-morbidities:**					
Cardiac	84	0,435	0,013*	0,226	0,840
Vascular	95	0,493	0,035*	0,256	0,952
**Year before treatment**					
Number of exacerbations in which treatment was needed	173	0,847	0,064	0,711	1,010
**In-hospital treatment**					
NIV	35	0,439	0,045*	0,196	0,983

N: number of patients; NIV: non-invasive ventilation.

The data used for this study was made publicly available at https://www.ebi.ac.uk/biostudies/studies/S-BSST870

## Discussion

In this retrospective study, we aimed to describe the clinical practice of HFNC-treatment in patients admitted with an AECOPD and to identify factors associated with success or treatment failure.

We observed that HFNC-treatment in patients with an AECOPD was initiated most frequently for sputum stasis (N = 69, 39.9% of all treated patients) and to relief symptoms of dyspnea (N = 46, 27% of all treated patients) as primary goal. In 61% of the patients treated the treatment was successful suggesting a possible role for HFNC-treatment in COPD-patients. HFNC-treatment was tolerated well, in only 17% of the treated patients treatment was stopped because it was not tolerated. This 17% is lower than the reported incidence of discomfort in treatment with NIV; 30–50% [8, 9]. This suggests that HFNC might be more easy to tolerate than NIV. This is supported by Jing et al. who reported that treatment with HFNC was better tolerated than treatment with NIV before [21].

To our knowledge this is one of the first large real-life studies investigating HFNC-treatment in a population with severe exacerbations of COPD. This study was also performed on the regular ward rather than on the ICU. In our study clinicians choose for HFNC most often because of sputum clearance problems. In most COPD-patients chronic sputum production as well as decrease in mucociliary clearance is a challenging problem [22–25]. By delivering warm and humidified air, the HFNC can facilitate secretion removal through optimal function of mucosa and increased water content in mucous [26, 27]. In a study by Hasani et al. treatment with humidified high flow resulted in a significant increase in mucociliary clearance in patients with bronchiectasis [26]. Which indicates that HFNC-treatment could play a role in treatment of COPD-patients and/or bronchiectasis with complaints of sputum stasis. Trials assessing sputum extraction however are difficult due to the limited availability of strong endpoints.

In our cohort, the presence of vascular and/or cardiac co-morbidities and treatment with NIV were independently associated with HFNC-treatment failure. We hypothesize that both factors are more common in more vulnerable or severely ill patients with an AECOPD influencing HFNC success rates.

Perhaps due to the fact that these co-morbidities often remain untreated. Heart failure for example can mimic or present itself concomitantly with an AECOPD [28], which makes proper treatment more difficult. Like multiple other studies our study suggests an urgent need for better assessment and treatment of co-morbidities in COPD-patients [29–31]. Next the HFNC itself can have a negative outcome on the co-morbidities, e.g. in the occurrence of cardiac arrhythmias [32].

Furthermore, patients who did not tolerate NIV (n = 15), often also not tolerated HFNC-treatment (N = 8) potentially due to the severity of respiratory failure. Moreover NIV-treatment is often needed in patients who are severely ill and are in need of reducing their work of breathing. As in NIV, HFNC is able to reduce work of breathing by reducing minute ventilation [16, 33]. Furthermore HFNC generates a slight positive end-expiratory pressure (PEEP), but far less than NIV. This PEEP has been shown to improve V/Q matching by improving recruitment of alveoli [34]. Multiple studies have assessed HFNC in comparison to NIV. These studies have shown that HFNC is non-inferior in reducing P_a_CO2 and that there was no difference in 30-day mortality, intubation-rate or treatment failure compared to NIV-treatment [35–38]. The difference in established PEEP between the two devices could be a factor in why HFNC-treatment also fails in patients in which NIV has been unsuccessful. Moreover due to the only slight generation of PEEP inspiratory demands of COPD-exacerbated patients may not be met In HFNC. Spoletini et al. stated that in patients with a high breathing workload and NIV might be more suitable than HFNC and HFNC might be best situated in between standard oxygen and NIV to threat hypoxemia and respiratory failure [39].

Our study suggests that HFNC-treatment might be less suitable for patients who received NIV-treatment as is seen in the multivariate logistic regression analysis. However no differences were seen between patients treated successfully and non-successfully when looking at step-down after NIV-treatment or NIV-failure as reason for HFNC-treatment (see Table 4). Only a slight difference was seen in progression to NIV (1 vs. 4), although numbers were small. Furthermore patients treated successfully were treated longer on average than patients with treatment failure. Although this was not an independent risk factor for treatment success.

The stable state lung function was not associated with a better or worse outcome of HFNC-treatment. FEV1 has been known to show high variability among individuals and therefore FEV1 might not be such a good predictor for outcomes during exacerbations [40, 41]. Potentially this might be different when hyperinflation is present during exacerbations, however data assessing hyperinflation were not available [42, 43].

In our study COPD-patients showed a significant reduction in respiratory rate and improved saturation after HFNC-treatment. This is consistent with a study by Jeong et al. in which an improved respiratory rate and saturation in hypercapnic patients (33 patients with AECOPD) was seen after HFNC-treatment [44]. The reduction of respiratory rate also suggests a reduction in work of breathing, as is also seen in other studies in COPD-patients [45, 46]. Successful treatment in this COPD-population seems depended on the improvement of these respiratory parameters, this is shown by the significant difference in respiratory rate and saturation between patient treated successfully and non-successfully during and after treatment (see Table 4).

Our study suggests that HFNC could also play a role in the treatment of AECOPD in patients in which O_2_-suppletion alone is not enough. As is also stated in a recent published practical guideline for the use of HFNC [47]. The guideline was not yet published when this study started, but recommends HFNC over conventional oxygen therapy in hypoxemic acute respiratory failure. A recommendation that this study supports considering the success rate of 61% in our patient population of COPD patients with respiratory failure. Although our study did not investigate the difference between treatment with HFNC versus conventional oxygen therapy.

Moreover, our study implies a possible role for HFNC in COPD-patients with sputum-related problems. As was stated before by Crimi et al. whom showed that HFNC was capable of increasing mucus production in patients with AECOPD and coexisting bronchiectasis [48]. NIV and other O_2_-suppletion are often less suitable for this indication due to the fact that the O_2_- or NIV-mask needs to be removed to clear sputum. Resolving sputum stasis might reduce the work of breathing as well; as it can reduce dyspnea and lower the respiratory rate and thereby work of breathing, but further research is needed to confirm this notion.

Our study has some limitations. This study is a retrospective study, performed in a single center without a control group. Therefore, it is difficult to completely value data about clinical efficacy. Furthermore, the group of patients was mixed as HFNC was used for several indications and aims. However, there were no differences in reasons for HFNC-treatment between the success and non-success group. Third, the definition of successful treatment could be debated as this was decided on judgement of the clinicians without strict criteria. Since this is a retrospective study a standard operating procedure was not available, we defined successful treatment as the absence of treatment failure combined with the clinical aim of treatment and whether this goal was achieved based on the judgement of the treating clinical physician to obtain the most objective outcome. The main advantage of such a clinical endpoint is however that it reflects actual care for this severely exacerbated COPD-patients. For future prospective studies we would recommend to predefine such criteria. Another weakness of this study is that arterial blood gasses analysis data is not available. During clinical practice these are not routinely performed during HFNC-treatment. This reflects the aims and position of the HFNC-treatment, since HFNC was not used as treatment for respiratory acidosis.

This large retrospective study showed that real world HFNC-treatment in patients with an AECOPD was initiated most often for sputum stasis as primary reason (N = 69, 40%), 61% of all patients ended the treatment successfully based on clinical judgement, and were treated longer. Factors associated with improved outcomes of HFNC-treatment was the absence of vascular and/or cardiac co-morbidities and a longer duration of HFNC-treatment. In clinical practice HFNC-treatment reduces respiratory rate and improves saturation in patients with a COPD-exacerbation. Based on the now reported results, future trials should consider to add mucus related outcomes to their designs, to address the apparent position of HFNC use in the clinic.
